# The state of methamphetamine (‘tik’) use among youth in the Western Cape, South Africa

**DOI:** 10.7196/SAMJ.2016.v106i11.10814

**Published:** 2016-11-02

**Authors:** E H Weybright, L L Caldwell, L Wegner, E Smith, J J Jacobs

**Affiliations:** 1 Department of Human Development, College of Agricultural, Human, and Natural Resource Sciences, Washington State University, Pullman, Washington, USA; 2 Department of Recreation, Park, and Tourism Management, College of Health and Human Development, The Pennsylvania State University, University Park, Pennsylvania, USA; HIV and AIDS Programme, University of the Western Cape, Cape Town, South Africa; and School of Biokinetics, Recreation and Sport Sciences, Faculty of Health Sciences, North-West University, Potchefstroom, South Africa; 3 Department of Occupational Therapy, Faculty of Community and Health Sciences, University of the Western Cape, Cape Town, South Africa; 4 Bennett Pierce Prevention Research Center for the Promotion of Human Development, The Pennsylvania State University, University Park, Pennsylvania, USA; and HIV and AIDS Programme, University of the Western Cape, Cape Town, South Africa; 5 HIV and AIDS Programme, University of the Western Cape, Cape Town, South Africa

## Abstract

**Background:**

Methamphetamine use among youth in the Western Cape Province of South Africa has increased at alarming rates over the past decade. Although current estimates of youth use exist, they range from 2% to 12%.

**Objectives:**

To identify (*i*) the prevalence of methamphetamine use in Western Cape youth and (*ii*) the association between use and known risk factors for methamphetamine use.

**Methods:**

Data were obtained from 10 000 Western Cape Province Grade 8 learners in 54 secondary schools (mean age 14.0 years). Prevalence was descriptively reported while risk factors for past-month use were modelled in a hierarchical logistic regression with demographic, socioeconomic status, substance use, sexual activity and relationship predictors.

**Results:**

Approximately 5% (*n*=496) of learners had used methamphetamine within their lifetime. Of these users, 65% (*n*=322) had used in the past month or week. Compared to never users, past-month users were more likely to be male, less likely to have a present or partially present mother, less likely to live in an apartment/flat/brick house, more likely to have used alcohol and tobacco and more likely to report having a same-sex partner.

**Conclusion:**

Results replicate previously known methamphetamine risk factors and highlight the need to address methamphetamine use in comprehensive prevention initiatives.

Methamphetamine (MA) use in South Africa (SA), especially in Cape Town and its surrounding areas, has been increasing at alarming rates. Locally known as ‘tik’ owing to the popping sound made when heated, the inexpensive and easily accessible nature of MA led to a surge in use in the early 2000s, primarily within the Western Cape Province.^[1]^ Recent evidence suggests MA use continues to be prevalent, second only to marijuana as the primary substance of abuse in patients seeking treatment.^[2]^ Although the proportion of youth seeking treatment primarily for MA use decreased between 2006 and 2011 to 25%, this does not reflect youth seeking treatment for polysubstance use where MA use is secondary to other substances.^[3]^ Targeting youth for treatment remains an effective approach to reducing use and preventing adult MA abuse. Broadly, MA use is associated with individual short-term negative outcomes such as psychosis and aggression, and long-term outcomes such as increased exposure to HIV. MA use also negatively affects society. For example, use has been associated with contraction and spread of communicable diseases such as TB, as well as domestic violence and degredation of community safety.^[1,4,5]^

Unfortunately, accurate estimates of MA use are difficult to obtain owing to factors such as difficulty accessing users and under-reporting of use. Because of these and other issues, estimates of SA youth MA use exist, but range between 2% and 12%,^[4]^ making it challenging to accurately identify current prevalence and subsequent tracking of prevalence over time. Having an understanding of whether MA use is changing and who is most affected can inform targeted prevention and intervention approaches. In addition, it can inform provision of healthcare and support services such as HIV education, because of the association between MA use and sexual risk behaviour.^[1]^

Prior research on MA use has suggested the use of large-scale school surveys to monitor prevalence and how use is associated with risk factors.^[4,6]^ However, few large-scale surveys have addressed youth MA use in detail and those that have collected youth data have focused on lifetime use, ignoring recency of use. Since 2005, four SA school survey studies have collected MA use data. Study details and prevalence findings are reviewed below.

In 2011, Morojele *et al*.^[6]^ surveyed 20 227 Grade 8 - 10 learners from eight Western Cape districts representing both metro and non-metro areas. Across all districts, 1.4% of Grade 8, 2.1% of Grade 9, and 2.6% of Grade 10 learners reported ever using MA in their lifetime. The 2008 SA Youth Risk Behaviour Survey^[7]^ collected lifetime MA use from 10 270 Grade 8 - 11 learners in nine provinces. Across all provinces, 8.1% of Grade 8 learners reported ever using MA in their lifetime. Prevalence was slightly higher for the Western Cape Province, with 9% of Grade 8 - 11 learners reportedly ever using MA in their lifetime. Two other school-based surveys were collected by Plüddemann *et al*.^[8,9]^ Data collected from 4 605 learners in the Cape Town Metropole in 2005 found 12.6% of Grade 9 learners had used MA in their lifetime.^[8]^ The second study from 2006 found 8.8% of Grade 8 - 10 learners (*n*=1 561) in the Cape Town South educational district had ever used MA in their lifetime.^[9]^

In these school-based surveys, secondary school MA use ranges from 1.4% (Grade 8 data) at the lowest to a high of 12.6% (Grade 9 data). From a chronological perspective, use has gone from 12.6% (2005 data), 8.8% (2006 data), 8.1% (2008 data), and finally to 1.4% (2011 data). What is not clear is whether these data are demonstrating an actual decline in MA use among school-attending SA youth, or whether variations in samples due to geographical location and grade representation are driving this trend. Moreover, there is little indication of recency of MA use and how use may be associated with risk factors, as these large-scale studies often only address lifetime prevalence.

A systematic review of MA risk factors in North American youth has identified being male, Caucasian, ever having sex, prior licit and illicit substance use, and reporting being in a homosexual relationship as associated with greater use.^[10]^ Research on SA youth MA use suggests these associations may hold cross-culturally. Within SA, studies have found youth MA use to be associated with poor mental health including aggression and depression,^[9]^ sexual activity including vaginal and anal sex,^[8]^ and poor academic attainment or school dropout.^[4]^

The current study is one of the few large-scale data collections from SA school-attending youth to address recency of MA use and its association with risk factors. Research questions focus on (*i*) the prevalence of MA use in the sample including lifetime, past-year, past-month, and past-week use and (*ii*) whether known risk factors for MA use identified by Russell *et al*.^[10]^ hold cross-culturally when comparing using and non-using youth.

## Methods

### Participants and procedures

Data were taken from the HealthWise SA trial testing varying implementation conditions across 56 high schools in the Metro South and Metro East Western Cape school districts.^[11]^ To determine which schools would be recruited, the SA team developed a matrix with schools' postal code, assessment of level of safety, assessment of level of access, total number of students enrolled in Grade 8 and Grade 9, number of classrooms in the school, school fees, and a poverty index based on the schools' locations. This information was used to develop a principal factor analysis procedure that assisted in the assignment to condition. Used in the current study, baseline data were collected in March 2012 from Grade 8 learners in 54 schools (mean standard deviation (SD) students per school 190.6 (63.8)) prior to curriculum implementation. Learners with missing data on all variables of interest (2% of sample) were removed, resulting in a final sample of 10 000 youth. Youth were 14 years old on average (SD 0.99), evenly split on gender (53% female), with 48% identifying as coloured (i.e. mixed ancestry), 43% black African, 7% white and 2% other. For comparison, the 2011 Western Cape census data reported 49% coloured, 33% black African, 16% white and 1% Indian.^[12]^ Institutional Review Board and Research Ethics Committee approval for the current study was obtained from study-affiliated universities and authorised by local education districts.

### Measures

Learners were asked about MA use, including one question for lifetime use: ‘How many times have you used tik in your life?’ and three separate questions for past year, month and week: ‘How many times in the past timeframe did you use tik?’

Risk factors were modelled after Russell *et al*.^[10]^ and made use of available data. These included demographic characteristics, socioeconomic status (SES), substance use, and sexual activity and relationship items.

Demographic variables included sex: ‘Are you a boy or a girl?’, race: ‘How do you identify yourself?’ with response options of black, white, coloured, Indian and other, and who the learner lived with using two questions of ‘During the last 6 months, has your mother/father lived with you?’ Response options included ‘no, my mother/father is dead,’ ‘no, none of the time,’ yes, some of the time,’ and ‘yes, always or almost always.’ Responses were dichotomised for parsimony, collapsing responses of ‘yes, some of the time’ and ‘yes, always or almost always’ and collapsing ‘no, none of the time’ and ‘no, my mother/father is dead.’

SES was measured dichotomously using home type with the item ‘Which of the following best describes your home?’ Responses categories of ‘shack,’ ‘wendy house or backyard building/room,’ ‘tent,’ and ‘other’ were combined to compare with response of ‘brick house, flat, or apartment.’

Substance use was measured using items of alcohol: ‘How many drinks of alcohol have you had in the past 30 days (month)?’ and tobacco: ‘During the past month, how many cigarettes have you smoked?’ Both items were dichotomised to reflect use and non-use in the past month.

Finally, sexual activity and relationship were measured with two items. Sexual activity was captured with the question ‘Have you ever had sex? This means intimate contact with someone during which the penis enters the other person.’ Relationship was measured from the item ‘Are you currently in a relationship?’ Same-sex relationship was calculated as a female learner responding ‘Yes, I have a girlfriend’ or a male learner responding ‘Yes, I have a boyfriend’, as opposed to learners reporting being in an opposite-sex relationship or no relationship.

### Analytic plan

Prevalence of MA use was categorised into mutually exclusive groups of lifetime (i.e. used in their lifetime but not in the past year, month or week), past year (i.e. used in the past year but not the past month or week), past month (i.e. used in past month but not in the past week), and past-week use with dichotomous yes/no responses. Categories of past month and past week were later collapsed when comparing past-month users with never users.

Prevalence was descriptively reported while risk factors for past-month and never use were modelled as outcomes in a hierarchical logistic regression where model A included demographic and socioeconomic status predictors and model B added substance use, sexual activity and relationship predictors to model A. This nested approach allowed for identification of predictors above and beyond those in model A using the likelihood ratio test. Differences in the −2 log-likelihood (−2LL) for model A were compared with model B using a χ^2^ test where a significant difference (using difference in degrees of freedom (df)) would indicate model B is a better fit to the data.

## Results

When examining the prevalence of MA use in the sample, 95.0% of youth reported no lifetime use, 1.3% used in their lifetime and not in the past year, 0.4% used in the past year and not in the past month, 1.2% used in the past month and not in the past week, and 2.0% used in the past week. Demographic characteristics of each user group are reported in Table 1.

Of the learners that reported any MA use (*n*=496), 26.4% (*n*=131, 95% confidence interval (CI) 22.1 - 29.9%) reported lifetime use and not past year use, 8.6% (*n*=43, 95% CI 6.5 - 11.5%) past year use and not past month use, 24.3% (*n*=121, 95% CI 20.2 - 27.8%) past-month use and not past-week use, and 40.5% (*n*=201, 95% CI 36.7 - 45.3%) past-week use.

Table 2 reports results of logistic regression nested models on past-month MA use (including past-month and past-week use, *n*=322) compared with never users reporting no lifetime use (*n*=9 504). Results from model A indicate that, compared with youth who have never used MA in their lifetime, past-month users were more likely to be male (OR 2.19); more likely to be black (OR 4.42), white (OR 5.72) or other (OR 3.98) than coloured; less likely to have a present or partially present mother/father than an absent or deceased mother/father (OR 0.37 and 0.77, respectively); and less likely to live in a brick house, apartment or flat (OR 0.46).

Model B demonstrated better fit indicated by significant likelihood ratio test (χ^2^ (4)=43.27, *p*<0.001). Compared with never users, past-month users were more likely to be male (OR 1.67); more likely to be black (OR 5.49) or white (OR 4.15) than coloured; less likely to have a present or partially present mother than an absent or deceased mother (OR 0.43); less likely to live in a brick house, apartment or flat (OR 0.51); more likely to have used alcohol (OR 4.01) and tobacco (OR 3.73) in the past month; and more likely to report having a same-sex partner (OR 8.82). When compared with model A, the presence or absence of the father, racial category of other, and ever having consensual sex were not significant predictors in model B.

## Discussion

This study is one of the few large-scale secondary school datasets capturing past year, month, and week MA use in Western Cape Province, SA. Results indicate that 4.9% of youth report lifetime MA use while 3.2% report past-month use. Given that youth and young adults comprised one-third of the population in the 2011 Cape Town census, current study results have the potential to translate into a large number of MA-using youth. In comparing with prior learners reporting lifetime prevalence use, the current study is the most recent data collection (compared with 2011^[6]^) and falls somewhere in the middle of previously reported youth lifetime MA prevalence, which ranged from 1.4% to 12.6%. The most similar sample can be taken from the study conducted by Morojele *et al*.,^[6]^ which found that the Western Cape Metro South and Metro East education districts (the same districts used in the current study) on average reported 2.1% lifetime use. However, the data provided spanned Grades 8 through to 10, making direct comparisons difficult. Across all eight education districts in Western Cape Province, 1.4% of Grade 8 learners used MA in their lifetime. ^[6]^ However, these eight school districts included both rural and urban settings while the current study was focused within the Metro, or urban, area only. Overall, when comparing current study results with available data, it remains challenging to address the inconsistencies in measurement and sampling to obtain accurate estimates of MA use. This suggests youth MA use remains a high priority within Western Cape Province. Going further, the current study finds a more concerning subgroup of past month and week users, making up 65% of learners who report using.

Analyses support prior SA work indicating MA risk factors are generally consistent with Western samples^[10]^ suggesting that although the context across North American countries and SA differs, risk factors for youth MA use may not. One notable exception is that the current results did not find that having consensual sex was associated with MA use. This finding is inconsistent with prior studies from the Cape Town area^[8]^ and may be due to the low rate of sexual activity for youth at this age (7.6% in the sample). Despite this, the urgency of addressing youth MA use may be especially important within Western Cape Province. For example, the SA context poses additional risks given the young age of users (14 years old on average), the prevalence of HIV risk in the area, and the fact that MA is inexpensive and easily accessible.

Demographic risk factor results also supported prior research finding individuals not consistently living with mother and father and with lower SES (as evidenced by home type and/or educational attainment as proxies) are more likely to use MA. Current results indicated that compared with coloured youth, black and white youth are more likely to use MA. This finding is inconsistent with prior community-based studies finding higher rates of MA use in coloured individuals.^[1]^ However, most of the school-based results have not reported MA use by race and greater integration is occurring within Western Cape secondary schools, representing more black and coloured, but also white, learners.

The logistic regression results highlight the continued need to address MA use in policy and broad prevention initiatives to address risk factors which are difficult to change such as gender, race, SES and household composition. Policy changes such as keeping schools open as recreation hubs in the afternoons and evenings would allow for the provision of supervised programming in low-income areas for youth who may have absent parents at home. In addition, community centres could be used for skill development programmes to promote youth development within a safe environment. Access to leisure and recreation opportunities is especially important for youth and is associated with delayed substance use initiation in Cape Town females.^[13]^

In addition, results suggest avenues for targeted approaches where youth could be screened for the presence of known predictors and recent (e.g. past month/week) use and then be provided with additional services. Implementing approaches such as SBIRT (screening, brief intervention and referral to treatment), which has been used in hospital settings,^[14]^ may be effective to quickly identify youth and direct them to existing services within the educational system to address modifiable risk factors.

Although the current study provides insights into SA youth MA use and associated risk factors that have not previously been identified, limitations exist. Firstly, due to the school-based sample, high-risk youth who have dropped out of school would not be captured within the current data, resulting in conservative estimates of use. However, all school-based studies experience this same issue. Secondly, the current study makes use of youth self-report data which are prone to self-report bias. Finally, results from the current study of Western Cape youth many not be generalisable to more rural areas. Even with these limitations, the current study provides important detail on the recency of Western Cape youth MA use and informs public policy and prevention approaches to address use.

## Figures and Tables

**Table 1:** Sample characteristics by MA use group

	No lifetime use (*n*=9 504, 95.0%), %	Used in lifetime and not past year (*n*=131, 1.3%), %	Used in past year and not in past month (*n*=43, 0.4%), %	Used in past month and not in past week (*n*=121, 1.2%), %	Used in past week (*n*=201, 2.0%), %
Gender					
Male	46.2	61.5	60.5	68.6	62.1
Female	53.8	38.5	39.5	31.4	37.9
Race					
Black African	41.0	67.2	72.1	71.1	66.0
Coloured	49.7	23.7	11.6	14.9	13.2
White	7.1	6.9	9.3	10.7	17.8
Other	2.2	2.3	7.0	3.3	3.1
Age (years), mean (SD)	13.9 (0.95)	14.5 (1.2)	14.8 (1.1)	15.1 (1.2)	14.9 (1.3)

*N*=10 000. MA use groups are mutually exclusive.

**Table 2:** Results of fitting hierarchical logistic regression models to MA use data*

	Model A	Model B
OR (95% CI)		
Male	2.19† (1.73 - 2.79)	1.67‡ (1.14 - 2.46)
Race (coloured serves as reference group)		
Black African	4.42† (3.17 - 6.17)	5.49† (3.31 - 9.11)
White	5.72† (3.74 - 8.76)	4.15† (2.15 - 8.01)
Other	3.98† (1.95 - 8.12)	NS
Living with mother (None of the time/deceased serves as reference group)		
Always/some of the time	0.37† (0.28 - 0.48)	0.43† (0.28 - 0.65)
Living with father (None of the time/deceased serves as reference group)		
Always/some of the time	0.77§ (0.61 - 0.98)	NS
Home type	0.46† (0.36 - 0.58)	0.51§ (0.35 - 0.74)
Past-month alcohol use		4.01† (2.72 - 5.90)
Past-month tobacco use		3.73† (2.45 - 5.68)
Ever had consensual sex		NS
Same-sex relationship		8.82† (5.68 - 13.71)
Goodness-of-fit		
−2LL	2 464.80	931.13
LR statistic	331.92	375.19
*n* parameters	7	11
*p*-value	<0.0001	<0.0001
AIC	2 480.80	955.13
LR test		Model A v. B
−2LL χ^2^		43.27† (df=4)

NS = main effect not significant; LR = likelihood ratio; AIC = Akaike information criterion.

* *N*=9 504. Modelled likelihood of using MA in past month compared with never using MA.

† *p*<0.001.

‡ *p*<0.01.

§ *p*<0.05.
